# Alternative mental health therapies in prolonged lockdowns: narratives from Covid-19

**DOI:** 10.1007/s12553-021-00581-3

**Published:** 2021-08-07

**Authors:** Petar Radanliev, David De Roure

**Affiliations:** Department of Engineering Sciences, University of Oxford, Oxford, OX1 3QG England, UK

**Keywords:** Alternative therapies, Art Therapy, Disease X, Covid-19

## Abstract

Identify and review alternative (home-based) therapies for prolonged lockdowns. Interdisciplinary study using multi-method approach – case study, action research, grounded theory. Only secondary data has been used in this study. Epistemological framework based on a set of digital humanities tools. The set of tools are based on publicly available, open access technological solutions, enabling generalisability of the findings. Alternative therapies can be integrated in healthcare systems as home-based solutions operating on low-cost technologies.

## Introduction

**Fig. 1:**
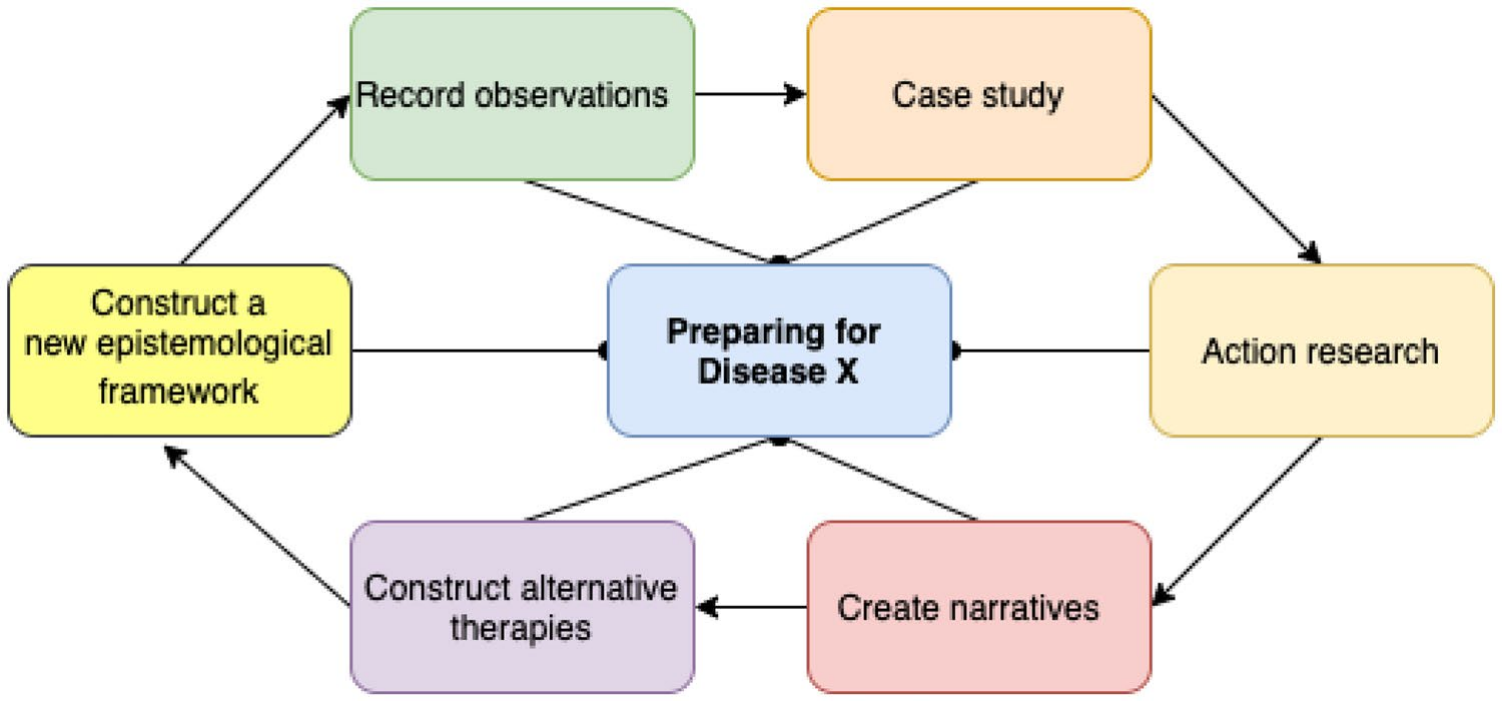
Research methodology—epistemological approach for studying Disease X events

Covid-19 has challenged the perception that humans are ‘autonomous and in control’ and exposed human vulnerabilities that threaten our social existence [1]. This article conducts an interdisciplinary study using multi-method approach (described further in Fig. 1) and data from Covid-19 to increase our preparedness for Disease X. Disease X is a term adopted by the World Health Organisation WHO where X stands for unexpected—and represents a hypothetical and unknown deadly and fast spreading pathogen. Disease X is simply a placeholder name, designed to promote research on classes of viruses and not on individual strains, with the aim of improving our preparedness to manage unforeseen pathogens. The term was coined in 2018, therefore Covid-19 can be considered as the first strain of Disease X. This project, however, refers to Disease X as the next—new and future pathogen.

The intent is to study extensively the case of Covid-19 and improve our preparedness for the next deadly and global pandemic. The next Disease X event can be as contagious as Covid-19 and deadly as Ebola (50–90% fatality) and could cause a pandemic similar to the filovirus that causes Ebola, it could be a virus that we already know, like the Nipah virus, it could be a completely new virus, or a frequent pathogen like the influenza virus. Disease X could be passed on from animals to humans, such as yellow fever, rabies, brucellosis, Lyme disease, or HIV, and could be even worse than Covid-19. The rising number of novel viruses is to a big extent caused by wildlife trade and ecological destruction of natural habitats, which cause the extinction of the larger predator animals, leaving small animals, like rats, bats and insects. Since the small animals can survive living with humans, when we change the ecology, they leave and move to urban areas. They can easily spread viruses in areas with low hygiene and high population density, which presents favourable conditions for viruses. Eventually some of these viruses reach a global hub (e.g., Wuhan).

Disease X can happen anytime, and we are not more prepared than we were for Covid-19, which will be remembered by politically motivated and uncoordinated responses. To prepare for Disease X, national healthcare policies need to support the adaptation of new technologies in healthcare systems to maximise the benefit from new digital solutions for pandemic management. Many developed countries—driven by Covid-19—have created national programs and initiatives enabling their healthcare systems to benefit from the increased digitalisation for pandemic management. But the adoption of new technologies is mirrored with an increased concern on the emerging risks from integrating such technologies in critical infrastructure – e.g., healthcare systems. These concerns are mostly related to security, privacy and trust, and can result with a lower rate of adoption. In addition, the less developed regions (e.g., Africa) have not been able to respond as fast to the Covid-19 challenges and are not getting the best benefit from new digital solutions.

### How can we adapt to future global pandemics?

The aim of this paper is to address existential questions emerging from the Covid-19 pandemic. The **first question** we ask is *how can we adapt to future global pandemics*? The Covid-19 pandemic has triggered an irreversible chain of events, reflecting new behaviours and changes in the way we do things. Some of these changes would have been impossible to imagine before Covid-19. Changes in handshakes, changing from fact-to-face and into online study, working from home, paying people to stay at home, among many other changes. This has resulted in a reality that is far different than just few months ago, and the world hasn’t adapted to this change, yet. We are currently experiencing a form of ‘state capitalism’, when the state authorises a substantial Keynesian stimulus by extending credit and making direct payments to businesses. Salary payments are calculated on the basis of the employee earlier creation of value in the market, rather than their current value or usefulness of their work. With the relatively short period of the Covid-19 pandemic, this has kept the markets operational. In a prolonged pandemic, state would have to take more radical actions, and in such prolonged widespread illness, the extra funds for people and hospitals would be much harder to sustain. Eventually, the system would need to return to normal, which would require further austerity, limiting the ability to respond to future pandemics. Such scenario leads only to a failed state and collapsed community and welfare system. The alternative is to continue with a form of state socialism, when the state continues to provide payments and protect markets to counter the effect of a prolonged pandemic. This also leads to economic recession and in a long run, failed state, collapsed community and welfare system. Because of these unattractive scenarios, we need to re-examine our perception of value as a subset of economics. This means that we need to examine the value of other aspects of value in our society (e.g., arts and humanities) and the values of alternative (i.e., home based) therapies for our mental wellbeing during prolonged lockdowns, in our efforts to building a more resilient society and economy. This presents a radically different approach to our perception of value and goes far beyond the traditional understanding of economic value in physical and mental healthcare.

### Why are we not prepared for Covid-19 and Disease X?

The world has faced many pandemics in the past,^1^ but Covid-19 has demonstrated that we remain unprepared. Research funding on the topic of global pandemics has historically been very limited and this is noticeable in the historical research output. A Web of Science search on the word ‘global pandemics’ resulted with just over 9,800 records (data from years 1900–2021). When I compare this with 397,074 records on business, 474,774 on fishing, 807,000 on ‘marketing’, then the lack of research problem becomes obvious. Research records on global pandemics have more than tripled since the rise of Covid-19, in a recent study published just after the first wave, the same Web of Science search on the word ‘global pandemics’ resulted with 3708 [2] (data from years 1900–2020). But this is hardly comparable to some of the data I found on other scientific records. While performing a very different search, we found 4,399 records on the topic of ‘Brexit’ – a term that was coined during a referendum in 2016. In other words, more research has been funded on Brexit (from years 2016–2021), than on global pandemics (data from years 1900–2020). This shows how desperately we need to re-examine our perception of value of research (on global pandemics). The underlaying cause for our unpreparedness for Covid-19 was the lack of funding (research), followed by the complexity of predicting which pathogen will be the cause of the pandemic and the complexity in observing, modelling and controlling the phenomenon.

### How can we adapt in anticipation of Disease X?

The first topic analysed in this study is related to **adapting** to pandemics and discoursing how qualitative methods can be applied to observe in what way different people adapted to Covid-19 and to capture digital narratives related to values from arts and humanities as alternative therapies for physical and mental health. In terms of relating values to economics, one of the hardest hit sectors during Covid-19 are directly related to arts and humanities (e.g., the creative industries, theatres, cinemas, visual and studio arts, performing arts). At the same time, these sectors have provided some of the crucial mental health support. To identify the real value of these sectors, a new epistemological approach (Fig. 1) is developed in this article, with unique tools to analyse the value of alternative therapies during Covid-19.

Recent studies on this topic discovered that ‘female healthcare workers are more vulnerable to mental health problems’ and that ‘timely and special care’ is required during such events [3]. Another recent study conducted a systematic review on what interventions are needed to address mental health issues in healthcare workers during infectious disease outbreaks [4]. The study grouped interventions into four categories: 1) informational support; 2) instrumental support; 3) organisational support; and 4) emotional and psychological support – in other words ‘psychoeducation and training, mental health support team, peer-support and counselling, therapy, digital platforms and tele-support’. Avoiding these requirements for timely and special care requirements, can result with a range of negative mental health effects. Although these effects need to be studied in much greater detail, recent studies are already investigating the links and the ‘causal influences of neuroticism on mental health and cardiovascular disease’ [5]. Other studies investigate the Covid-19 related mental health issues in college students [6]. Reports on other fields e.g., STEM advise that ‘a change in policies of organizations.. may be required to provide accommodation and compensation to minimize the negative impact on the professional status and career of men and women who work in STEM fields [7]. The research methodology in Fig. 1 building upon recommendations from recent studies that argue for using digital solutions in healthcare for online cognitive behavioural therapies and counselling services and mental care provision, while cautioning about the potential cyber risks [8]. A qualitative *case study* and *ethnographic* approach is used, to seek *narratives* to challenge our understanding of value. The aim of this article is to progress towards increased preparedness and increased understanding of managing global pandemics with emerging technologies. In particular, the study objectives are to identify alternative (home-based) therapies for prolonged lockdowns and record as much data on the values of arts and humanities in the Covid-19 pandemic, and apply multi-method research to use the data for increased preparedness for Disease X. As we have seen from Covid-19, the world needs to increase in efforts towards achieving a better control of managing fast spreading deadly pandemics.

This study integrates the distant fields of healthcare and arts and humanities. The research methodology applied grounded theory and was designed with an iterative methodology. Lessons learned from each iteration are used in the design and control of the next iteration cycle. To reduce and overcome the complexity of the iterative methodological process, and to progress towards a better understanding of the outcome of each iteration, a variety of complimentary but different techniques are used e.g., case study, action research. The complementary approach provided insights on the Disease X phenomenon at different lengths and time scales.

## How can we prepare for Disease X?

The first approach for preparing for a global disease analysed in this article is related to applying new technologies for managing the negative effects on healthcare systems and people’s mental health of prolonged lockdowns. This part of the study applies grounded theory with qualtitative case study research, to construct digital narratives related to physical and mental health during prolonged lockdowns, including home educational strategies and coping mechanisms, and to investigate alternative home-based mental health therapies. The aim is to devise alternative mental health solutions to prepare for prolonged social isolation caused by global pandemics. The analysis in this part of the research is based on recording observations on how humans around the world adapted to the Covid-19 pandemic. Through experimental observations the study progressively captures the complexity of adapting to global pandemics, while taking into consideration a number of parameters related to physical and mental health. The experimental observations cycle is reflexed in the *first postulate P*_*1*_: we can adapt to global pandemics.

During the catastrophic social changes caused by Covid-19, one of the hardest hit sectors in the first and the second wave are the creative industries, theatres, cinemas, and many other areas that belong to the arts and humanities sectors, such as visual and studio arts, performing arts. At the same time, these sectors have provided much of the essential mental health support. For example, most people coped with the Covid-19 lockdown by turning to Netflix and other home-based visual entertainment, mostly produced by performing artists in visual studios. In addition, arts and crafts were used as an educational strategy and coping mechanisms by parents during the lockdown [9]. Art therapy was found helpful with finding control in the Covid-19 chaotic times [10]. This defines the rationale for the first objective (O)_1_ of this article – which is to construct a new epistemological approach (i.e., method) for recording the real value of arts and humanities during Covid-19.

## Eistemological model for recording knowledge on preparing for Disease X

**Fig. 2:**
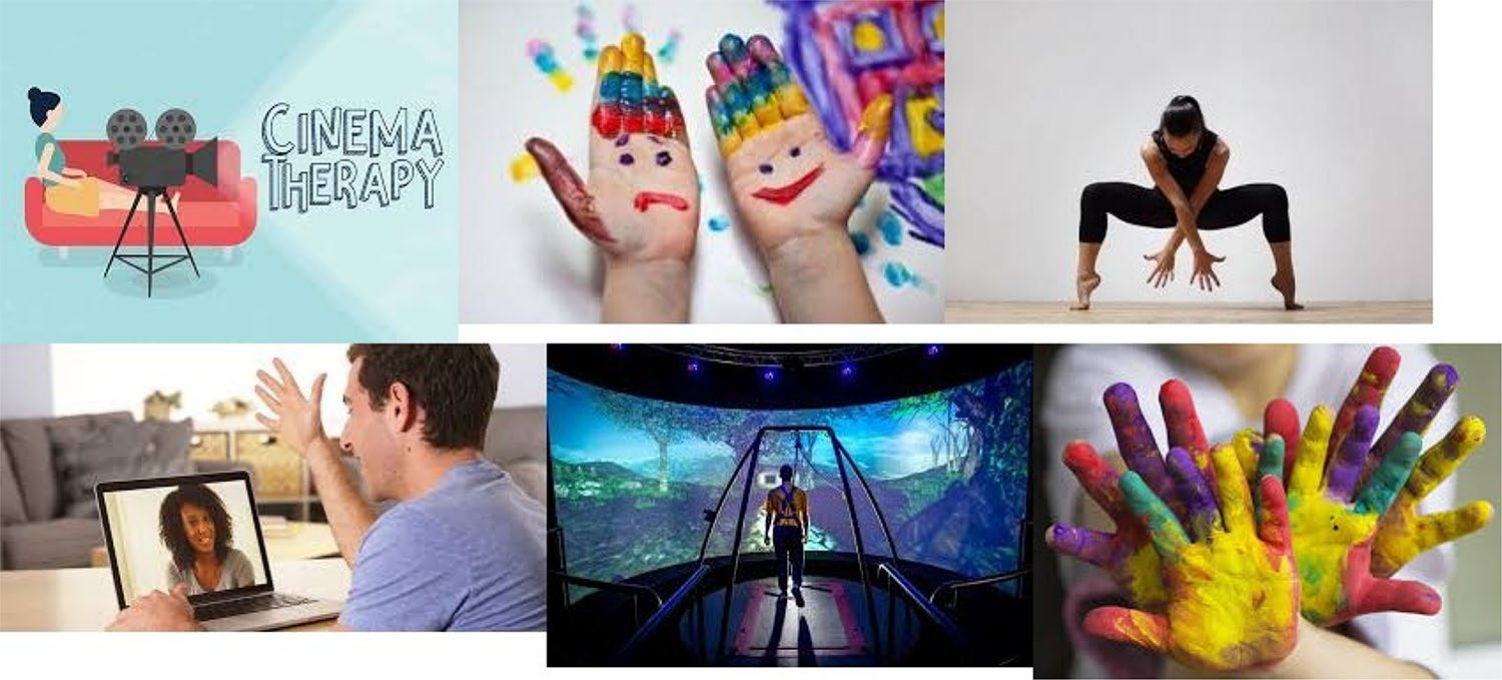
Alternative mental therapies for prolonged lockdowns

A combined case study, grounded theory, and action research methodology was the method of choice used to create digital narratives as a form of digital storytelling. The aim was to digitalise stories and to create a permanent records from everyday aspects of people life’s during Covid-19, presenting narratives as stories of values (and the struggles) in difficult times. The objective was to construct narratives of different methods (M) centred on arts and humanities as alternative **therapy (**Fig. 2**)** during prolonged lockdowns. The methods analysed include M_1_: cinema therapy [11]; M_2_: film/video-based therapy [12, 13]; M_3_: therapeutic filmmaking [14]; M_4_: Virtual Reality [15]; M_5_: video art (remix) therapy [16]; M_6_: psychotherapy [17–19]; M_7_: expressive arts therapy [20]; and M_8_: creative arts therapies (art therapy) [21, 22]; M_9_ dance/movement therapy [23–25]; M_10_ drama therapy [26, 27]; music therapy [28]; M_11_: poetry therapy [29–31], and psychodrama [32].

Some of the data sources (for the case study—action research) on how these methods are applied during Covid-19 included: (1) The Patient Voices digital stories; (2) Silence Speaks; (3) The Voice Library; (4) Cognitive Dynamics – bringing Art to Life; (5) online forums and blog posts; (6) Google Trends data; (7) consumer report data; (8) social media. The experimental developments emerging from the digital narratives is centred of specific dissemination approach (Table 1) found in different sources and used for constructing a particular point of view e.g. narratives of struggle, courage and transformation. There are however concerns in recent studies on the increased recreational use of virtual reality and the impact on physical and mental wellbeing during the Covid-19 lockdown [33]. This can be a concern, especially for vulnerable individuals. This was one of the reasons why this study applied the case study—action research, which facilitated observing and analysing the application of methods in different data sources and recording narratives on Covid-19 (collaborative action research). The case study – grounded theory research enabled creating narratives and complimented the analysed methods with a new set of epistemological tools (Et). The new set of tools provides a structure on how different methods (e.g., research films recordings) can become socially interactive, by being published online in open access for public dissemination, promoting and provoking interest for further exploitation (i.e., applying) of these methods during a Disease X event. The new set of epistemological tools (Et) was developed based on the value measured from each therapy during Covid-19 (i.e., popularity, acceptance, usability). The set of epistemological (i.e., digital humanities) tools (Et) and the values is enhanced with a conceptual epistemological framework (Ef), developed specifically to emphasise the values of different therapies – in a specific case scenario (e.g., global reach, targeted reach).

**Table 1:** Conceptual epistemological framework emerging from digital narratives on arts and humanities as therapies during global pandemics

ED	Exploitation (E) and dissemination (D_i_) of healthcare narratives to different audiences (collected during Covid-19)	Et	M
D_i1_	*YouTube* videos for global reach of personal narratives during Covid-19 e.g., the case of cinema therapy^2^	Et_1_	M_1_
D_i2_	*Embedded video recordings* on selected webpages for targeted reach of personal narratives during Covid-19 e.g., film/video-based therapy	Et_2_	M_2_
E_1_	*Viddler* community building through therapeutic filmmaking during Covid-19 for informing, interacting and engaging with local communities	Et_3_	M_3_
E_2_	Virtual reality of 3D shaped Covid-19 narratives with *SecondLife for Education*—for safe and secure real-time collaboration and virtual learning in a global community	Et_4_	M_4_
D_i3_	*Blogger* to publish narratives from video art (remix) therapy during Covid-19 and to encourage a discussion on their therapeutical values	Et_5_	M_5_
E_3_	Storytelling with digital humanities tools: *Omeka* sharing digital collections and creating media-rich online exhibits, with complex narratives that allow users to tag favourites and measure the value of psychotherapy	Et_6_ Et_1_	M_6_
E_4_	Data visualisation with digital humanities tools *Gephi*, and *Voyant* to present data visualisations of narratives on expressive arts therapy	Et_7_	M_7_
D_i4_	*Piktochart* for creating engaging content in different formats and translating complex data into a visual story with digital humanities tools on narratives of creative arts therapies (art therapy)	Et_8_	M_8_
E_5_	*WeVideo* for video creation and editing with digital humanities tools to inspire meaningful example-based learning on the topic of dance/movement therapy	Et_9_	M_9_
E_6_	*TikTok* for online presence in new and emerging media—short 60 s videos; followed with poster papers in *QQ*, *WeChat*, and *Printterest*; reshared as news in *Reddit*, and run as verbatim with text on *Tumblr*, and *Medium*,—on the topic of drama therapy, music therapy	Et_10_	M_10_
D_i5_	*Scribd*, *SlideShare*, *SlideRocket*, *authorSTREAM*, *present.me*, and other less know platforms e.g., *Zooniverse*, *Web Observatory* for sharing of narratives on the topics of poetry therapy, and psychodrama	Et_11_	M_11_

^2^https://www.youtube.com/channel/UCCYX4s1DCn51Hpf1peHS30Q

The epistemological framework undertakes experimental developments in researching the: (a) need to integrate digital narratives of different arts and humanities therapies with the digital humanities tools (listed in Table 1); (b) need to integrate arts and humanities therapies and existing reciprocities on digital narratives. The expected difficulties include the difference in duration of Disease X and the timeline of Covid-19 – presenting a different set of difficulties, e.g., disabling action research and the ability to participate and influence the outcomes. Alternatives to mitigate this risk include observing narratives from past events (recorded online) and suggesting potential alternative therapies as solutions during a Disease X event. This means that the case study – action research will change at some point during the therapy – from active engagement, to passive observation i.e., grounded theory.

## Conclusion

This study developed solutions for alternative therapies that can be applied with low-cost mobile devices for managing metal health during a Disease X event—while preserving the patients security, privacy and trust. The article presented a conceptual epistemological framework based on digital humanities tools that can be used for alternative therapies during prolonged lockdowns. The epistemological framework is designed through studying narratives and applying grounded theory, case study and action research on qualitative data samples from Covid-19. The expectation is that the epistemological framework will assist developing countries in harnessing the value of new technological solutions. The framework can be applied in anticipation of a Disease X event, to manage and enable the creation of national initiatives for integrating alternative therapies in healthcare systems, as solutions operating on low-cost technologies.

### Limitations and future directions

Although the solutions are aimed at developing and developed countries, the development and validation of the solutions was primarily be applied in developed countries—using the existing knowledge and secondary data on digital healthcare systems. This was grounded on the secondary data availability, which was found mostly from technologically advanced healthcare systems i.e., in developed countries. The technological solutions and initiatives for alternative therapies developed in this project need to be supported with testbeds, which can be costly to build and developed countries are faced with financial constraints. The study identified some of the most suitable world leading testbeds (i.e., UK digital catapults), and real-world digital healthcare projects (Table 1) that could be used for testing and validating the new alternative therapies – after the current lockdown restrictions have ended. Alternative (i.e., suitable and similar) digital testbeds exist and are operational in the EU, UK and USA, and shortage of testbeds is not expected to be a concern. By developing and testing the solutions in state of the art testbeds, the framework will maximise the value for developed countries and enable developing countries to benefit from tested and verified digital solutions for Disease X management.

## Data Availability

All data and materials included in the article.

## Footnotes

1 https://en.wikipedia.org/wiki/Pandemic
